# Increased risk of corneal transplant rejection following SARS-CoV-2 infection and vaccination

**DOI:** 10.1007/s10792-026-04261-x

**Published:** 2026-09-09

**Authors:** Natalie E. Allen, Rachael L. Niederer, Akilesh Gokul, Jie Zhang, Anna Casey, Georgia Tippett, Charles N. J. McGhee

**Affiliations:** Department of Ophthalmology, Faculty of Medical & Health Sciences, Aotearoa-New Zealand National Eye Centre, University of Auckland, Private Bag 92,019, Auckland, New Zealand

**Keywords:** Cornea, Infection, COVID-19, Vaccination, Rejection, Corneal transplant

## Abstract

**Background:**

To determine the impact of COVID-19 infection and vaccination on risk of corneal transplant rejection.

**Methods:**

All corneal transplants performed in Auckland, New Zealand (NZ), between January 2010 and December 2022 were included. Demographic, clinical, and surgical characteristics were recorded, along with COVID-19 vaccination and infection history. Time to corneal transplant rejection was analysed using Weibull survival regression with clustered standard errors at the graft level to account for repeated events and time-varying covariates.

**Results:**

A total of 1183 corneal transplants were performed in 926 patients, with a median age of 52 years (IQR 32–70); 47.5% were female. The median follow-up was 11.7 years ± 3.8. Within 3 months following COVID-19 vaccination, 1.1% of corneal transplants were rejected; within the same period following COVID-19 infection, 2.3% of transplants rejected. Overall, 277 transplants (23.4%) experienced rejection. On multivariable analysis, COVID-19 vaccination within 3 months was associated with elevated rejection risk (HR 1.834, *p *= 0.012), while COVID-19 infection within 3 months carried the greatest risk (HR 3.533, *p *< 0.001). Corneal transplant rejection was associated with significantly worse visual acuity follow-up (rejection median 20/160, no rejection 20/70, *p* < 0.001).

**Conclusions:**

COVID-19 vaccination and infection were independently associated with an increased likelihood of corneal transplant rejection, with infection conferring the greatest risk. These findings suggest that systemic immune activation induced by SARS-CoV-2 immunisation may disrupt the delicate balance of transplant immunity and tolerance. This is the first large cohort study demonstrating an independent association between recent COVID-19 infection, vaccination, and increased risk of corneal graft rejection.

## Introduction

Severe acute respiratory syndrome coronavirus 2 (SARS-CoV-2, COVID-19) was the largest pandemic of the twenty-first century, with 779 million cumulative cases to date [1]. The pandemic reached its peak in 2021, resulting in significant healthcare challenges related to both access and the virus itself. The pandemic was declared to no longer be a “global health emergency” by the World Health Organization on May 6th, 2023 [1, 2]. This was due in part to the development and distribution of recombinant mRNA vaccines, of which over 13 billion doses have been administered globally [1]. Although the widespread use of vaccination has been pivotal in controlling the pandemic, both vaccination and COVID-19 infection can elicit immune activation that can occasionally become dysregulated. Such responses have been implicated in ocular inflammation, with increased risk of recurrent or de novo uveitis and scleritis [3].

A corneal transplant is the definitive treatment for many sight-threatening conditions and offers the possibility of fully restoring vision. It is the most frequently performed tissue transplant worldwide after skin grafting [4]. In New Zealand (NZ), the number of corneal transplants performed is around 6.1/100,000 population/year, a rate that has been steadily increasing over the past two decades [5–7]. Success of corneal transplantation has historically been attributed to the relative immune privilege of the cornea, which confers relatively low rates of rejection [8, 9]. However, when rejection does occur, the clinical consequences are profound. A single episode may jeopardize graft clarity, leading to irreversible vision loss and the need for repeat transplantation [10]. Each failed corneal transplant impairs quality of life and visual function, but also increases the risk of rejection in subsequent transplants, and contributes to greater healthcare burden and donor tissue demand [11, 12]. Preventing rejection and identifying patients at the highest risk, therefore, remains central to improving long-term outcomes and safeguarding vision.

Viral infections and systemic immune responses are recognized as important risk factors for rejection in corneal transplants and solid organ transplant populations [13]. Cytomegalovirus (CMV) in particular is reported to approximately double the risk of rejection in kidney, liver, and lung transplants. Adenovirus, parvovirus, and herpes simplex virus have also been associated with increased rejection risk. The mechanisms proposed include systemic inflammation, bystander immune activation, and direct endothelial injury, all of which also occur in COVID-19 infection and vaccination [14–16]. Despite extensive global experience with COVID-19, knowledge regarding its influence on corneal transplant rejection is derived largely from small studies and anecdotal case reports [17–19]. This uncertainty complicates counselling for corneal transplant recipients, who must weigh the largely unknown potential risks of infection and vaccination, at a time when our understanding of COVID-19-related immune dysregulation remains incomplete. This study aims to address this deficit by examining the relationship between COVID-19 vaccination, COVID-19 infection, and corneal transplant rejection in a large cohort of patients undergoing corneal transplantation in Auckland, NZ.

## Methods

Local ethics committee approval was granted by the Health and Disability Ethics Committee of New Zealand (study number 2023 EXP 18674). All methods were performed in accordance with the Declaration of Helsinki.

All patients who underwent corneal transplantation in Auckland, NZ, from January 2010 to December 2022, both in private and public settings, were included. This data therefore included patients of nine different corneal surgeons across three surgical sites. All corneal tissue was supplied via the New Zealand National Eye Bank based in the University of Auckland. Data collection occurred between January 2024 and July 2024, with all transplants having at least one-year postoperative follow-up at the time of data collection.

Clinical and demographic data were obtained through a combination of hard copy and electronic clinical records. Variables collected included patient demographics, graft type, visual acuity, clinical features at presentation, COVID-19 vaccination (Pfizer-BioNtech) dates, and infections. Additional patient-related factors, such as the number of unattended appointments and treatment adherence, were also recorded. “Compliance concerns” were defined as any documented issue with ocular medication adherence, including not using prescribed eye drops, running out of medication, or documented confusion regarding the dosing regimen. A rejection episode was defined when an endothelial rejection line was present or when there was inflammation (stromal infiltrate, keratic precipitates, cells in the anterior chamber, or ciliary injection) involving a graft that was previously clear. The time interval post vaccination or infection event was set at a maximum of 90 days as immunological changes post vaccination persist until 90 days [20]. Adaptive immune activation following SARS-CoV-2 infection is most dynamic during the first weeks to months after infection, encompassing the period of evolving humoral and cellular immune responses. Although immunological memory persists well beyond three months, a 90-day interval has also been widely adopted in COVID-19 research as a clinically meaningful time window and is the conventional threshold used to distinguish reinfection from persistent infection [21, 22].

## Statistical analysis

Data was collected and analysed in Stata version 15 (STATACorp 2017, College Station, TX). Categorical data were reported as n (%) and continuous as median (interquartile range [IQR]). Time to graft rejection was modelled using parametric survival regression with a Weibull distribution to accommodate the observed decline in hazard over time since graft. The analysis accounted for potentially multiple episodes of rejection per graft, and time-varying covariates were incorporated to reflect changes in exposure to COVID-19 infection and vaccination over follow-up. To account for within-graft correlation across multiple time intervals, we compared two modelling approaches: a Weibull survival model with robust standard errors clustered at the graft level, and a Weibull model incorporating a gamma-distributed frailty term. The Weibull clustered model demonstrated a lower AIC/BIC and a higher log-likelihood than the shared frailty model, indicating superior model fit, and was therefore retained for the primary analyses. Model fit was assessed, and the model with clustered standard errors at the graft level demonstrated superior fit and was therefore selected as the final model. Although the Weibull model supports both accelerated failure time and proportional hazards interpretations, we report hazard ratios (HR) for interpretability. All tests were two-sided, and a *p*-value of ≤0.05 was considered significant.

## Results

During the study period, 1183 corneal grafts were performed in 926 patients. Median follow-up was 11.664 years ± 3.803. Subject demographics are reported in Table 1. Median age at transplantation was 52 years [IQR 32–70] and differed between types of graft, with a median age of 45 (penetrating keratoplasty), 31 (anterior lamellar keratoplasty), and 71 (endothelial keratoplasty) (*p* < 0.001), respectively. Sex was male in 486 (52.5%) and female in 440 (47.5%). Self-reported ethnicity included Caucasian (52.3%), Pacific peoples (20.3%), and Māori (17.9%). A quarter of all patients had documented medication compliance concerns.

**Table 1:** Demographics of corneal transplant recipients over study period

	N=1183 grafts 926 patients
Age at transplantation	Median 52 years (IQR 32–70)
Female	440 (47.5% of 926)
*Ethnicity*	
Caucasian Pacific people Māori Asian MELAA Unknown	484 (52.3% of 926) 188 (20.3%) 166 (17.9%) 62 (6.7%) 19 (2.1%) 7 (0.8%)
*Type of corneal transplant*	
Penetrating Lamellar Endothelial	719 (60.8% of 1183) 126 (10.6%) 338 (28.6%)
Documented compliance concerns	306 (25.8% of 1183)
COVID-19 vaccination	660 (71.3% of 926)
COVID-19 infection	314 (33.9% of 926)

COVID-19 vaccination was received by 660 patients (71.3%) with a total of 2290 COVID-19 vaccinations given during the study period. There was an average of 2.7 vaccinations received per patient, with one initial dose and up to 5 subsequent boosters. There was a median interval of 134 days between doses. COVID-19 infection was documented in 314 patients (33.9%) with a total of 431 COVID-19 infections occurring during the study period. A total of 26 allograft rejection episodes were documented within three months following 2290 COVID-19 vaccinations, corresponding to a cumulative incidence of 1.1% over the three-month period. These post-vaccination rejections represented 3.9% of all corneal grafts in the study that experienced rejection within the same time frame**.** A total of 10 rejection episodes were documented within three months following 431 COVID-19 infections, corresponding to a cumulative incidence of 2.3% over the three-month period.

Graft rejection occurred in 277 (23.4%) corneal grafts during the study period. Risk factors for corneal graft rejection are reported in Table 2. On univariable analysis, increasing age at the time of graft was protective, with each additional year reducing the hazard of rejection (HR:0.970, *p* < 0.001). Ethnicity demonstrated marked differences in rejection risk: compared with Caucasian recipients, Māori (HR:2.286, *p* < 0.001) and Pacific peoples (HR:2.764, *p* < 0.001) were at significantly higher risk, while Asian recipients did not differ significantly (HR:1.559, *p *= 0.134). Type of graft also influenced outcomes, with endothelial grafts (HR:0.393, *p* < 0.001) associated with a lower risk relative to penetrating keratoplasty. Non-compliance with treatment was strongly associated with rejection (HR: 3.333, *p* < 0.001). Notably, COVID-19 infection (HR:3.358, *p *= 0.001) within the preceding three months was associated with increased risk.

**Table 2:** Risk factors for corneal transplant rejection. *P*-values less than 0.05 have been bolded

	Univariable		Multivariable	
	HR	*P* value	HR	*P* value
Age at graft	0.970	** < 0.001**	0.990	**0.017**
Female	1.046	0.738	1.095	0.509
*Ethnicity*				
Caucasian Māori Pacific peoples Asian	Reference 2.286 2.764 1.559	NA **< 0.001** **< 0.001** 0.134	Reference 1.330 1.706 1.555	NA 0.161 **0.003** 0.149
*Transplant type*				
Transplant type Penetrating Anterior lamellar Endothelial	Reference 0.803 0.393	NA 0.289 **< 0.001**	Reference 0.648 0.737	NA **0.039** 0.153
Compliance concerns	3.333	** < 0.001**	2.782	** < 0.001**
Covid vaccine within 1 month	1.227	0.773	1.186	0.810
Covid infection within 1 month	2.925	0.065	2.991	0.060
Covid vaccine within 2 months	1.028	0.921	1.179	0.553
Covid infection within 2 months	2.614	**0.021**	2.742	**0.016**
Covid vaccine within 3 months	1.565	0.055	1.834	**0.012**
Covid infection within 3 months	3.358	**< 0.001**	3.533	**< 0.001**

Note: Bold is statistically significant

In the multivariable model, many of these associations persisted after adjustment for confounders. Age at graft remained protective (HR:0.990, *p *= 0.017 95% CI: 0.832–1.22), translating to a 9.6% reduction in hazard of rejection by decade. Ethnic disparities persisted, with Pacific peoples continuing to demonstrate a significantly elevated risk of rejection compared with Caucasians (HR:1.706, *p* = 0.003 95% CI: 1.611–1.899), while the increased risk in Māori (HR:1.330, *p *= 0.161 95% CI: 1.072–1.522) recipients was no longer statistically significant. The graft type with a statistically significant lower risk became anterior lamellar (HR:0.648,* p* = 0.039 95% CI: 0.428–0.763) instead of endothelial. Compliance concerns continued to show a strong association with rejection (HR:2.782, *p* < 0.001 95% CI: 2.446–2.808). Both COVID-19 vaccination (HR:1.834, *p* = 0.012 95% CI: 1.801–1.899) and recent COVID-19 infection (HR:3.533, *p* < 0.001 95% CI: 3.356–3.764) within 3 months were associated with increased risk of graft rejection.

Median visual acuity at last follow-up was 20/80 [IQR 20/40 to count fingers], and there was significantly worse visual acuity in those with a history of corneal transplant rejection (rejection median 20/160, no rejection 20/70, *p* < 0.001). No significant difference was observed in visual outcome after treatment for rejection in those with corneal graft rejection following COVID-19 vaccination (*p* = 0.404) or infection (*p* = 0.548) compared to rejection episodes occurring at other times.

## Discussion

This study examined corneal transplant rejection in 1183 corneal transplants over a 12-year period, including the COVID-19 pandemic. We observed corneal transplant rejection in 1.1% of grafts in the three months following COVID-19 vaccination, and in 2.3% of patients following COVID-19 infection within three months. COVID-19 vaccination was associated with a 1.834 increased hazard of rejection, and COVID-19 infection with a 3.533 increased hazard of rejection on multivariable analysis.

The literature specifically addressing COVID-19 infection and vaccination in relation to corneal transplant rejection remains limited to mainly case reports and case series [17, 19, 23, 24]. Most reported cases of rejection following vaccination have occurred in penetrating keratoplasties and were generally mild and reversible with treatment [17, 25, 26]. However, one large study across two centres in Italy and England found no increased incidence in corneal transplant rejection during the COVID-19 vaccination period [27]. However, this study examined rates of graft rejection against regional vaccination rates and did not examine risk at an individual level following vaccination. A smaller study of 77 cases of transplant rejection found no significant increase in graft rejection during the “risk period” following COVID-19 vaccination, defined as the subsequent 60 days [28]. Reports of graft rejection following COVID-19 infection are more sparse, limited to just a handful of case reports with temporal association [18, 29, 30]. This is the first study to demonstrate a statistically significant association between corneal transplant rejection and both recent COVID-19 vaccination and infection.

The proposed mechanism of action of corneal transplant rejection appears similar for COVID-19 vaccination and infection. Acute COVID-19 is characterized by a profound inflammatory response, with elevations in proinflammatory cytokines and widespread immune activation [14]. Adaptive immunity primed through mimicry, bystander activation, and endothelial injury makes donor tissue a more potent immune target and may cause rejection [8, 15, 17]. This also occurs following vaccination, though to a lesser extent [15]. COVID-19 infection activates all inflammatory proteins, whereas the vaccine activates only the spike (S) protein. Therefore, the antibody and T-cell response is targeted only towards spike (S) following vaccination, which is much larger and broader following COVID-19 infection [31, 32]. This may explain the increased risk of rejection following an infection compared to vaccination, a finding that is mirrored in other ocular sequelae. Uveitis flares occur after both COVID-19 vaccination and infection, but have a higher incidence following infection. A study of 235,228 individuals found that the incidence of uveitis flare post vaccination was 1.18 (95% CI: 1.13–1.22), whilst the incidence following COVID-19 infection was 1.61 (95% CI: 1.37–1.89) [33]. This is also true for optic neuritis. A study of 1.7 million individuals found that the risk of developing optic neuritis following COVID-19 vaccination was reduced compared to COVID-19 infection (RR of 0.09, 95% CI, 0.07–0.12, *P* < 0.001) [34].

There is a longstanding link between viral infections and solid organ transplant rejections; COVID-19 is no exception. Cytomegalovirus, adenovirus, herpes simplex virus, parvovirus, and enterovirus are all implicated in the rejection of kidney, lung, and liver transplants [13]. The association between COVID-19 infection and transplant rejection is also observed in these groups. Kidney transplant recipients with prior COVID-19 infection have been shown to face significantly increased risks of acute rejection on biopsy, even in the absence of direct viral invasion of the graft [35]. Rejection episodes following COVID-19 vaccination have also been reported in kidney, lung, liver, and pancreas transplants, although the majority of these are case reports [36, 37]. This, in turn, explains the significantly increased mortality with COVID-19 infection for transplant recipients. A systematic review of kidney transplant recipients with COVID-19 reported an average mortality rate of 23% across 74 studies. [38] However, solid organ recipients differ significantly from corneal transplant recipients as the former require extensive immunosuppression [11, 39].

These findings highlight that vaccination or COVID-19 infection may serve as an immune trigger for corneal transplant rejection. COVID-19 infection appears to be a greater risk, particularly by 90 days post infection, and more likely to cause complications in those who are unvaccinated.^34^ Therefore, clinicians ought to discuss with patients the balance between the risk of vaccination compared with its net benefit. Patients should be well informed of the symptoms of corneal graft rejection to ensure prompt presentation should this occur.

In addition to examining the role of COVID-19 infection and vaccination, this study suggests that multiple demographic and clinical factors contribute to the risk of corneal transplant rejection. Increasing age at the time of transplant was associated with a decreased risk of corneal graft rejection (HR: 0.990). Recipient age at the time of corneal graft is recognized as a central determinant of survival [10, 40–42]. Anterior lamellar keratoplasty demonstrated a protective effect (HR:0.648). This aligns with previous work describing lower immunogenicity of anterior lamellar transplants compared with full-thickness procedures, a consequence of the relative immune privilege of endothelial tissue [40, 41, 43]. Ethnicity emerged as a major factor in graft outcomes, with Pacific peoples (HR:1.706) experiencing greater rates of graft rejection compared with NZ Europeans. This may be a result of systemic barriers and socioeconomic deprivation [44, 45]. Documented compliance concerns also substantially increased the risk of corneal transplant rejection (HR:2.782 *p* < 0.001), in keeping with literature suggesting increased graft complications with poor medication compliance [46–48].

The current study provides unique insight into the potential link between COVID-19 and corneal graft rejection, but it must be appreciated in the context of its limitations. This study required retrospective data collection and, therefore, relied on clear documentation for accurate data. Risk factors for graft rejection such as neovascularisation, socioeconomic status and reason for graft were unable to be analysed. Detection bias may have influenced the observed associations, as corneal transplant recipients may have undergone more frequent clinical assessment during the COVID-19 pandemic, increasing the likelihood of detecting graft rejection episodes despite diagnosis being based on established clinical criteria. The impact of vaccination may also be masked due to clinicians recommending prophylactic treatment. COVID-19 infection documentation required a positive test processed by a NZ laboratory or self-reported to the Ministry of Health. If an individual did not test for the virus or did not report their infection, the episode was not recorded by our database. This is particularly relevant later in the pandemic as infection numbers rose and reporting became less mandated. This likely resulted in non-differential exposure misclassification, which would be expected to bias hazard ratio estimates towards the null, suggesting that the observed association between COVID-19 infection and corneal graft rejection may represent a conservative estimate of the true effect. It is estimated based on global serological testing from 2020–2022 that 59.2% (95% CI: 56.1%–62.2%) of individuals have been infected with COVID-19, which is far greater than the 35.9% recorded in this study, though New Zealand had more strict and extensive lockdown periods than most countries [49]. The potential underreporting and undertesting of COVID-19 infection means that the true impact of infection on corneal transplant rejection is likely underestimated. However, the records of COVID-19 vaccination are likely to be more accurate as reporting to the Ministry of Health is mandatory.

COVID-19 infection and vaccination represent novel, independent risk factors for corneal transplant rejection. Among all demographic and clinical variables examined, COVID-19 infection emerged as the most significant predictor of rejection. However, the absolute rejection rate was low at 1.1%, despite statistical significance. For clinicians, this emphasises the need for vigilance during periods of heightened immune activation, whether from viral infection or immunisation. Furthermore, while both vaccination and infection can act as triggers, these events are relatively rare compared with the overwhelming protective benefits of vaccination in reducing morbidity and mortality from COVID-19. Prophylaxis guidelines are required for topical steroid use in corneal transplants in the peri-vaccination period. Future research should prioritise large, population-based analyses to clarify the magnitude of risk associated with both vaccination and infection, as current literature is dominated by case series and retrospective studies. Immunological profiling studies examining the pathways linking COVID-19 to alloimmune responses in the cornea may also be critical.

## Data Availability

This data is not available due to confidentiality.
